# Oral 4-(*N*)-stearoyl gemcitabine nanoparticles inhibit tumor growth in mouse models

**DOI:** 10.18632/oncotarget.21264

**Published:** 2017-09-23

**Authors:** Caixia Wang, Yuanqiang Zheng, Michael A. Sand oval, Solange A. Valdes, Zhe Chen, Dharmika S. Lansakara-P, Maolin Du, Yanchun Shi, Zhengrong Cui

**Affiliations:** ^1^ Inner Mongolia Medical University, School of Basic Sciences, Inner Mongolia Key Laboratory of Molecular Biology, Hohhot, Inner Mongolia, China; ^2^ Inner Mongolia University, Research Center for Laboratory Animal Sciences, Hohhot, Inner Mongolia, China; ^3^ The University of Texas at Austin, College of Pharmacy, Division of Molecular Pharmaceutics and Drug Delivery, Austin, Texas, USA; ^4^ Inner Mongolia Medical University, School of Public Health, Hohhot, Inner Mongolia, China

**Keywords:** plasma pharmacokinetics, oral bioavailability, tumor growth inhibition, immunohistostaining

## Abstract

In spite of recent advances in targeted tumor therapy, systemic chemotherapy with cytotoxic agents remains a vital cancer treatment modality. Gemcitabine is a nucleoside analog commonly used in the treatment of various solid tumors, but an oral gemcitabine dosage form remain unavailable. Previously, we developed the 4-(*N*)-stearoyl gemcitabine solid lipid nanoparticles (GemC18-SLNs) by incorporating 4-(*N*)-stearoyl gemcitabine (GemC18), an amide prodrug of gemcitabine, into solid lipid nanoparticles. GemC18-SLNs, when administered intravenously, showed strong antitumor activity against various human and mouse tumors in mouse models. In the present study, we defined the plasma pharmacokinetics of gemcitabine when GemC18-SLNs were given orally to healthy mice and evaluated the antitumor activity of GemC18-SLNs when given orally in mouse models of lung cancer. In mice orally gavaged with GemC18-SLNs, plasma gemcitabine concentration followed an absorption phase and then clearance phase, with a T_max_ of ~2 h. The absolute oral bioavailability of gemcitabine in the GemC18-SLNs was ~70% (based on AUC_0-24 h_ values). In mice with pre-established tumors (i.e. mouse TC-1 or LLC lung cancer cells), oral GemC18-SLNs significantly inhibited the tumor growth and increased mouse survival time, as compared to the molar equivalent dose of gemcitabine hydrochloride or GemC18 in vegetable oil or in Tween 20. Immunohistostaining revealed that oral GemC18-SLNs also have significant antiproliferative, antiangiogenic, and proapoptotic activity in LLC tumors. Formulating a lipophilic amide prodrug of gemcitabine into solid lipid nanoparticles may represent a viable approach toward developing a safe and efficacious gemcitabine oral dosage form.

## INTRODUCTION

Cancer is the second leading cause of death globally [1]. Chemotherapy remains a vital cancer treatment modality. Chemotherapeutic agents are generally administered intravenously or orally. Compared to intravenous injection, oral administration of cancer chemotherapeutic agents is more convenient and comfortable to patients and may favorably modify the safety, pharmacokinetics, dosing regimen, and ultimately the efficacy of anticancer drugs [2]. Moreover, there is evidence that although oral anticancer drugs are costly, the overall costs of oral chemotherapy tend to be lower than intravenous infusion [3, 4]. Finally and unique to the U.S., a combination of the under-reimbursement of oncology infusion services by the Medicare Program and the increasing Medicare coverage of oral anticancer drugs provides incentives for both physicians and patients to choose oral anticancer drugs [5, 6]. However, despite the aforementioned advantages, oral anticancer drugs currently on the market make up only a few percent of all available anticancer drugs, largely due to the lack of availability of bioequivalent oral dosage forms of anticancer drugs. For example, gemcitabine (2′,2′-difluorodeoxycytidine; dFdC), a nucleoside analog, is commonly used in monotherapy or combination therapy of various solid tumors, including non-small lung, ovarian, breast, and pancreatic cancer, but an oral gemcitabine dosage form remains unavailable. Veltkamp et al. (2008) evaluated the toxicity, tolerability, pharmacokinetics, and preliminary antitumor activity of gemcitabine in patients with advanced or metastatic cancer and found that systemic exposure of orally administered gemcitabine was low, with an estimated bioavailability of 10% only [7]. It was concluded that the extensive first-pass metabolism of gemcitabine to 2′,2′-difluorodeoxyuridine (dFdU) in the liver needs to be overcome to successfully deliver gemcitabine by the oral route [7].

Previously, there has been effort to increase the oral bioavailability of gemcitabine using pharmaceutical chemistry approach (e.g. prodrug). Examples of gemcitabine prodrugs tested include the LY2334737 (an amide derivative of gemcitabine), CP-4126 (also known as CO-101, an ester derivative of gemcitabine), SL-01 (another amide derivative of gemcitabine, i.e. 3-(dodecyloxycarbonyl)pyrazine-2-carbonyl gemcitabine)), and amino acid derivatives of gemcitabine (as a substrate to the PEPT1 transporter) [2, 3, 8]. LY2334737 is a valproate amide prodrug of gemcitabine synthesized by conjugating gemcitabine in the N^4^-position with valproic acid [9], and oral LY2334737 has been tested in multiple phase 1 clinical trials [10–14]. CP-4126 is a gemcitabine ester prodrug synthesized by conjugating gemcitabine in the 5’ position with elaidic acid, and oral CP-4126 has been tested in clinical trials as well [15–20]. Squalenoyl gemcitabine (SQdFdC) is another amide prodrug of gemcitabine synthesized by conjugating gemcitabine in the N^4^ position with 1,1′,2-tris-norsqualenoic acid, and the resultant SQdFdC self-assemblies to nanoparticles of 100-300 nm in water [21, 22]. Oral SQdFC showed potent antitumor activity in a rat leukemia model [8, 23]. Besides the SQdFdC self-assembled nanoparticles, other nanoparticles have also been tested for oral administration of gemcitabine. For example, gemcitabine was incorporated into poly (lactic-*co*-glycolic) acid (PLGA) nanoparticles, and data in a rat model showed that oral gemcitabine-in-PLGA-nanoparticles increased the systemic exposure of gemcitabine by over 21-fold, relative to oral gemcitabine alone, in spite of the rapid release of gemcitabine from the nanoparticles (i.e. 100% in 2 h *in vitro*) [24]. D07001-F4 is another gemcitabine nanoparticle formulation prepared in a self-microemulsifying drug delivery system, and an absolute oral gemcitabine bioavailability of 34% was reported in a nude mouse model using the D07001-F4 [25].

Previously, we developed 4-(*N*)-stearoyl gemcitabine solid lipid nanoparticles (i.e. 4-(*N*)-GemC18-SLNs or GemC18-SLNs) by incorporating 4-(*N*)-stearoyl gemcitabine (i.e. 4-(*N*)-GemC18 or GemC18), an amide prodrug of gemcitabine, into solid lipid nanoparticles prepared with soy lecithin, glycerol monostearate (GMS), Tween 20, and phospholipid derivative(s) of polyethylene glycol (2000) [26]. GemC18-SLNs, when given intravenously, were significantly more effective than the molar equivalent dose of free gemcitabine or GemC18 alone (i.e. in Tween 20) in controlling tumor growth in mouse models of lung or pancreatic cancers, and can overcome tumor cell resistance to gemcitabine as well [26–28]. Our GemC18-SLNs integrate the aforementioned prodrug and nanoparticle approaches together, prompting us to test their antitumor activity when given orally in mouse models in the present study.

## RESULTS AND DISCUSSION

Previously, we developed the 4-(*N*)-GemC18-SLNs by incorporating GemC18, a lipophilic amide prodrug of gemcitabine, into solid lipid nanoparticles prepared with soy lecithin, GMS, Tween 20, and 1,2-distearoyl-*sn*-glycero-3-phosphoethanolamine-N-[amino (polyethylene glycol)-2000] (i.e. DSPE-PEG_2k_) [26]. When given intravenously, GemC18-SLNs were significantly more effective than the molar equivalent dose of free gemcitabine or the GemC18 prodrug in inhibiting tumor growth in mouse models of lung cancer or pancreatic cancer, and the GemC18-SLNs can overcome tumor cell resistance to gemcitabine as well [26–28]. Other lipophilic amide prodrugs of gemcitabine such as LY2334734 and SQdFdC were reported to have a stronger antitumor activity than the molar equivalent dose of free gemcitabine when administered orally in animal models [9, 23], and oral LY2334734 also led to a higher systemic exposure of gemcitabine than oral free gemcitabine in a mouse model [9]. GemC18 is a lipophilic amide prodrug of gemcitabine; it is poorly soluble in water, with a solubility of 1.38 ± 1.60 μg/mL (vs. > 2 mg/mL for LY2334734) [9, 29]. Therefore, we tested the antitumor activity of GemC18-SLNs when given orally in mouse models. The size the GemC18-SLNs was 98 ± 10 nm, and their zeta potential was about -46 mV. As we previously reported, the encapsulation efficacy of the 4-(*N*)-GemC18 in the GemC18-SLNs is close to 100% [26]. Initially, we evaluated the stability of the GemC18-SLNs in an environment similar to that in the gastrointestinal (GI) tract, i.e. in non-fed simulated gastric fluid (SGF) and simulated intestinal fluid (SIF). When GemC18-SLNs were incubated in SIF (pH 6.8) for 6 h, their particle size did not significantly change (data not shown). In SGF (pH 1.2), however, the particle size increased by ~15% after 1 h of incubation, but did not further change thereafter (data not shown). Previous studies by Tobio et al. (2000) showed the presence of PEG or Poloxamer on the surface of nanoparticles helps to stabilize the nanoparticles and hinder their aggregation in gastric fluid [30]. Our GemC18-SLNs are PEGylated, and the PEG chains on the surface of the nanoparticles may have helped to prevent severe aggregation of them in the SGF. The release of GemC18 from the GemC18-SLNs was slow in both SGF (~17% in 2 h) and SIF (~15% in 6 h). The release beyond 6 h was not monitored, because data from a previous study showed that the GI transition time of orally gavaged charcoal-carried ^99m^Tc-DTPA in mice was 6-8 h [31]. Of course, since the GI tract has plenty of enzymes that can catalyze lipid degradation, the release rate of the GemC18 from the GemC18-SLNs in mouse GI tract is expected to be significantly higher.

**Figure 1:**
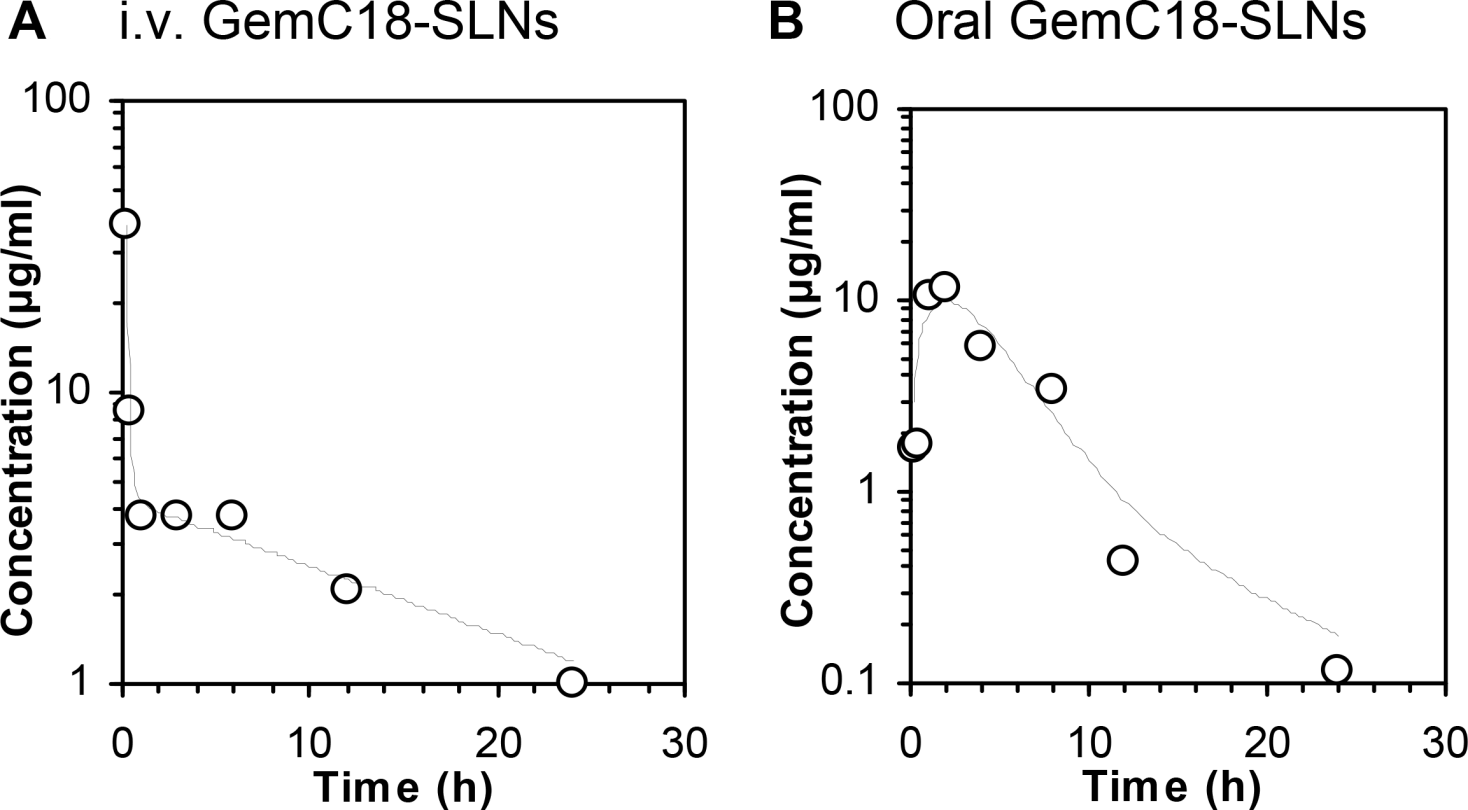
Plasma gemcitabine concentration (μg/mL) in BALB/c mice at different time points (h) after GemC18-SLNs were intravenously injected (**A**) or orally gavaged (**B**) into mice. The dose of GemC18 was 1 μg per mouse. Data shown at each time point are mean (○) from 3 mice (*n* = 3), and the curves were generated using PKSolver.

The plasma pharmacokinetics of gemcitabine in mice (i.e. in healthy BALB/c mice at 1 mg of GemC18) orally or intravenously dosed with the GemC18-SLNs were evaluated and compared to determine the oral bioavailability of gemcitabine in GemC18-SLNs. As shown in Figure 1A, the plasma gemcitabine level in mice intravenously injected with GemC18-SLNs appeared to fit a two-compartment model with an area under curve (AUC_0→24 h_) of 157 μg x h/mL. In contrast, the plasma gemcitabine level in mice that were orally gavaged with the GemC18-SLNs followed an apparent adsorption phase and then clearance phase, with a T_max_ of ~2 h and an AUC_0→24 h_ value of 110 μg x h/mL (Figure 1B). Additional pharmacokinetic parameters are listed in Table 1. The absolute oral bioavailability of gemcitabine in the GemC18-SLNs was calculated to be around 70%, based on the AUC_0→24 h_ values. Date from a previous study showed that the oral bioavailability of free gemcitabine was only ~10% in human subjects [7], and the absolute oral bioavailability of gemcitabine in female FVB mice (dose, 0.1 mg/kg) was reported to be about 45% [32]. Clearly, formulating gemcitabine into our GemC18-SLNs increased its oral bioavailability. In the present study, we determined the plasma concentration of gemcitabine, instead of GemC18. Therefore, the extent to which the gemcitabine was absorbed as GemC18 or as gemcitabine is unknown. It is also unknown exactly how the GemC18 in the GemC18-SLNs was absorbed into the blood circulation after oral gavage. It is thought that nanoparticles can be absorbed by enterocytes and microfold (M) cells in the small intestine [33–36], but Hu et al. (2016) reported that data in their studies did not support the direct absorption of intact solid lipid nanoparticles that were orally administered to rodents [37]. Of course, GemC18 may be released from the GemC18-SLNs while in the GI tract and then absorbed directly similar to the absorption of the other lipids in diets. In addition, some gemcitabine may also be hydrolyzed from the GemC18 in the GI tract and then absorbed as free gemcitabine, although to a very limited extent, as the oral bioavailability of gemcitabine was reported to be low [7, 32]. More experiments will have to be carried out to elucidate the mechanisms underlying the GemC18-SLNs’ ability to increase the systemic exposure of gemcitabine when administered orally.

**Table 1:** PK parameters after GemC18-SLNs were given to mice intravenously or orally

Intravenous			Oral		
Parameter	Unit	Observed	Parameter	Unit	Observed
k_10_	1/h	2.21	T_max_	h	2.02
k_12_	1/h	5.62	C_max_	μg/ml	17.56
k_21_	1/h	0.19	t_1/2α_	h	1.39
t_1/2α_	h	0.09	t_1/2β_	h	6.84
t_1/2β_	h	13.02	t_1/2Ka_	h	1.29
C_0_	μg/ml	434.34	AUC_0-24 h_	μg x h/ml	109.22
V	(mg)/(μg/ml)	0.002	AUMC	μg x h^2^/ml	659.78
Cl	((mg)/(μg/ml))/h	0.004	MRT	h	5.87
V_2_	(mg)/(μg/ml)	0.06	V/F	(mg)/(μg/ml)	0.02
Cl_2_	((mg)/(μg/ml))/h	0.01	Cl/F	((mg)/(μg/ml))/h	0.0146677
AUC_0–24 h_	μg × h/ml	156.66	V_2_/F	(mg)/(μg/ml)	0.01
AUMC	μg × h^2^/ml	2690.01	Cl_2_/F	((mg)/(μg/ml))/h	0.00015
MRT	h	13.69			

**Figure 2:**
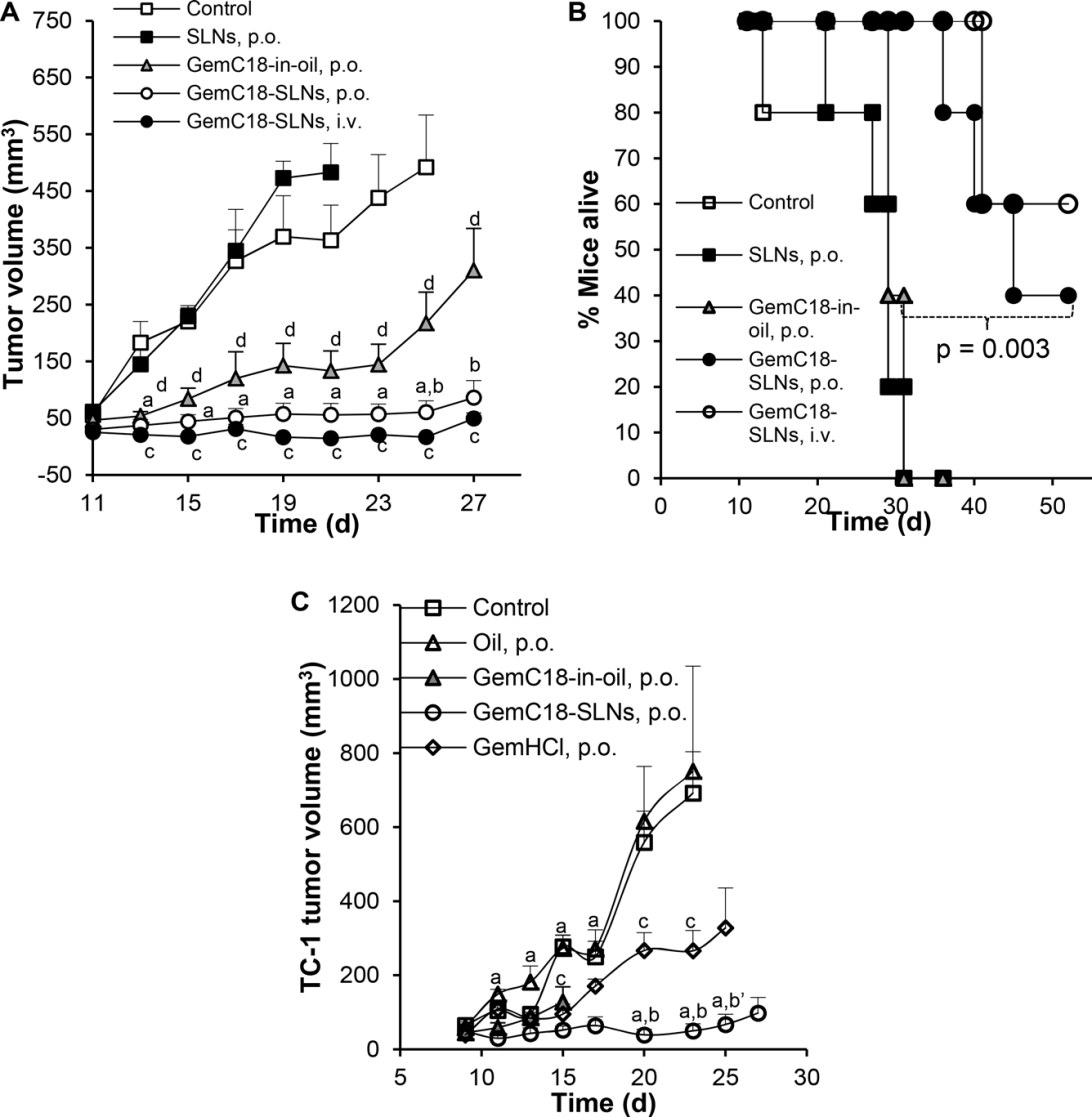
Antitumor activity of oral GemC18-SLNs against TC-1 tumors in a mouse model Shown are TC-1 tumor growth curves (**A**, **C**) and mouse survival curves (**B**). C57BL/6 mice were s.c. injected with TC-1 tumor cells on day 0. Starting on day 11, mice were randomized and orally gavaged with GemC18-SLNs, GemC18 in vegetable oil (GemC18-in-oil), or gemcitabine HCl (GemHCl). As controls, mice received vegetable oil alone (oil, p.o.), GemC18-free SLNs (SLNs, p.o.), GemC18-SLNs (i.v.), or left untreated. The dose of GemC18 in A and B was 250 μg/mouse/dose for the p.o. route (once daily), and 500 μg/mouse/dose for the i.v. route (twice a week). In C, the dose of GemC18 was 150 μg/mouse/dose (once daily), 85 μg/mouse/dose for GemHCl (i.e. molar equivalent to GemC18). Data shown are mean ± S.E.M. (*n* = 4–5). In A, ^a^ *p* ≤ 0.05, GemC18-SLNs (p.o.) vs. SLNs (p.o.) or Control; ^b^ *p* ≤ 0.05, GemC18-SLNs (p.o.) vs. GemC18-in-oil (p.o.); ^c^ *p* ≤ 0.05, GemC18-SLNs (i.v.) vs. Control; ^d^ *p* ≤ 0.05, GemC18-in-oil vs. Control. In B, *p* = 0.003, GemC18-SLNs, p.o. vs. GemC18-in-oil, p.o. (Log-rank Mantel-Cox test). In C, ^a^ *p* ≤ 0.05, GemC18-SLNs (p.o.) vs. Oil (p.o.) or Control; ^b^ *p* ≤ 0.05, GemC18-SLNs (p.o.) vs. GemHCl (p.o.); ^b’^ *p* = 0.059, GemC18-SLNs (p.o.) vs. GemHCl (p.o.); ^c^ *p* ≤ 0.05, GemHCl (p.o.) vs. Control. Data for GemC18-in-oil (p.o.) after day 15 were not reported because 2 out of 5 mice died after day 15.

The antitumor activity of oral GemC18-SLNs was evaluated in two different mouse models of lung cancer, mice with TC-1 or LLC tumors. Previously, we reported that TC-1 tumor cells are sensitive to the cytotoxicity of gemcitabine, GemC18, and GemC18-SLNs (i.e. IC_50_ values of 10.6 ± 1.1, 18.5 ± 1.7, and 41.4 ± 3.7 nM, respectively, 3 × 10^3^ cells, 48 h of incubation) [28]. In cell culture, gemcitabine and GemC18 were relatively more cytotoxic than GemC18-SLNs [26, 28], but in tumor-bearing mouse models, GemC18-SLNs, when given intravenously, were significantly more effective in inhibiting tumor growth than the molar equivalent dose of gemcitabine or GemC18 [26, 28]. In fact, GemC18 dissolved in a Tween 20 solution at the dose(s) tested did not show any significant antitumor activity in mice with TC-1 tumors or B16F10 tumors [26, 38]. Previously, Brusa and colleagues reported that peritumoral injection of GemC18 in a Tween 80 solution in mice with subcutaneously implanted HT-29 tumor cells did not significantly affect the tumor growth [39]. In our previous studies in mice with pre-established TC-1 tumors or BxPC-3 tumors, mice were i.v. injected with the GemC18-SLNs at 1 mg/mouse, 9 or 13 days apart (i.e. 2 mg of GemC18 in 11 days on average) [26]. Therefore, based on the absolute bioavailability value of ~70%, in the first efficacy study, mice with TC-1 tumors were orally gavaged with GemC18-SLNs at 250 μg GemC18/mouse daily. In mice with TC-1 tumors, tumor grew aggressively if left untreated (Figure 2A). Oral GemC18-SLNs significantly inhibited the tumor growth, whereas oral GemC18-free SLNs did not show any significant activity (Figure 2A–2B), demonstrating that the SLNs as a carrier or delivery system for GemC18 were not active against TC-1 tumors, and that it was the GemC18 in the GemC18-SLNs that exerted the antitumor activity. However, it was the SLNs that enabled the GemC18 to strongly inhibit the tumor growth, because GemC18 in vegetable oil (i.e. GemC18-in-oil) given at the same dose and dosing schedule was not as effective as in the GemC18-SLNs in inhibiting the tumor growth (Figure 2A). In addition, mice that were orally dosed with the GemC18-in-oil did not survive as long as those orally dosed with the GemC18-SLNs (*p* = 0.003, Log-rank Mantel-Cox Test) (Figure 2B). In fact, some mice that were orally gavaged with the GemC18-in-oil exhibited significant weight loss and signs of distress (e.g. arched back, hair loss, and hypoactivity) during the treatment and had to be euthanized preemptively.

Another animal study was carried out in mice with TC-1 tumors to (i) understand whether the observed side effects of the GemC18-in-oil was caused by the vegetable oil and (ii) compare the antitumor activity of the GemC18-SLNs to that of free gemcitabine. The oral dose of GemC18-SLNs was reduced to 150 μg GemC18/mouse daily, because at 250 μg of GemC18/mouse daily, GemC18-in-oil was not well tolerated in the study above. Two of the five mice orally gavaged with GemC18-in-oil died after day 15. Mice that were orally gavaged with the vegetable oil alone did not exhibit any adverse reactions or reactions similar to that in mice orally gavaged with the GemC18-in-oil, indicating that it was the GemC18-in-oil formulation, not the vegetable oil, that caused the observed adverse effects. This finding also demonstrates the effect of different formulations of the same compound on the efficacy and toxicity of the compound in an animal model. Finally, oral gemcitabine hydrochloride (HCl) also inhibited the TC-1 tumor growth, as compared to when the tumor-bearing mice were left untreated, but was significantly less effective than the GemC18-SLNs at the molar equivalent dose of gemcitabine (i.e. GemC18, 150 μg/mouse vs. gemcitabine HCl, 85 μg/mouse) (Figure 2C).

**Figure 3:**
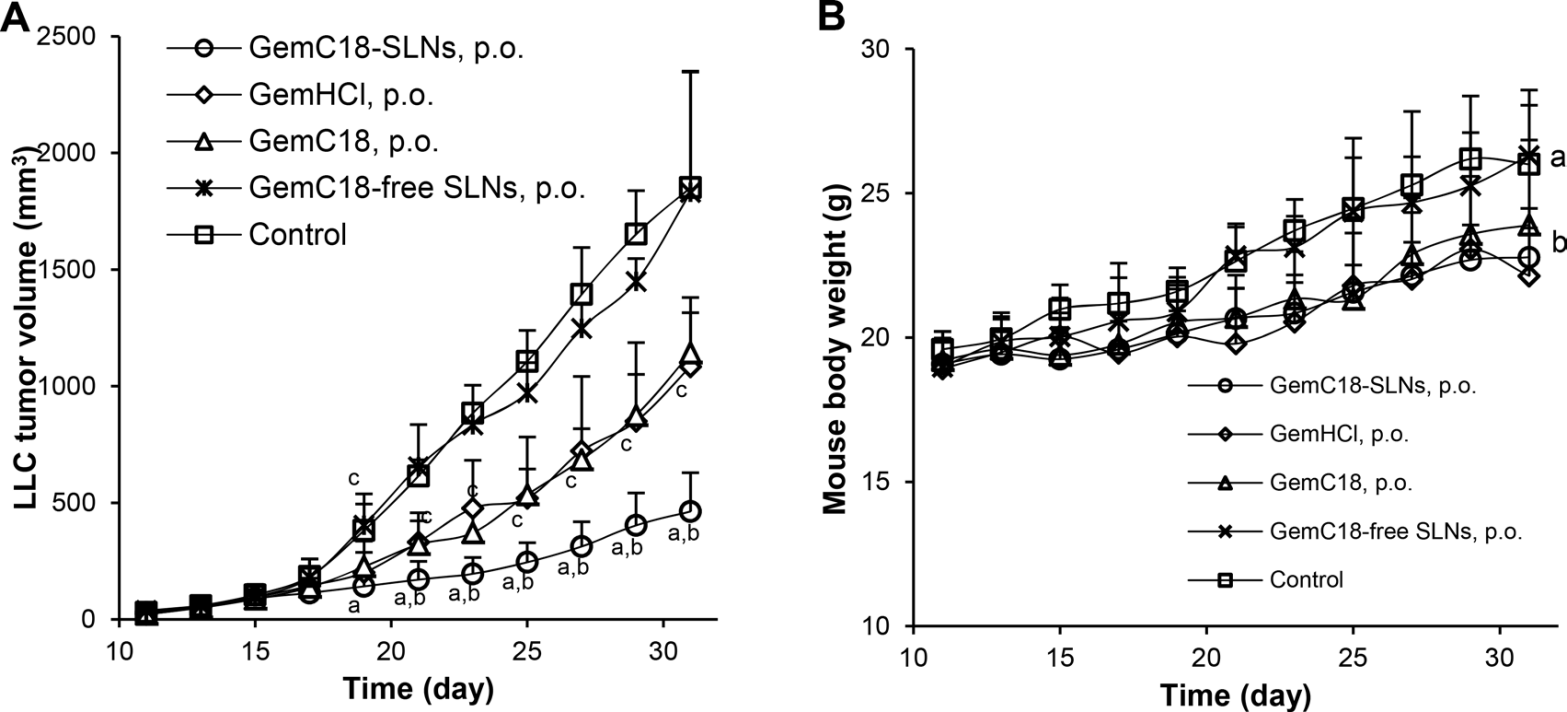
Antitumor activity of oral GemC18-SLNs against LLC tumors in a mouse model Shown are LLC tumor growth curves (**A**) and mouse body weight changes (**B**). C57BL/6J mice were s.c. injected with LLC tumor cells on day 0. Starting on day 11, mice were randomized (*n* = 8) and orally gavaged with GemC18-SLNs, GemC18 in Tween 20, or gemcitabine HCl (GemHCl), all in a 5% (w/v) mannitol solution. As controls, mice received GemC18-free SLNs (p.o.) or a mannitol solution (5%, w/v). The dose of GemC18 was 250 μg/mouse/dose (once in every two days), 141.5 μg/mouse/dose for GemHCl (i.e. molar equivalent to GemC18). Data shown are mean ± S.D. In A, ^a^ *p* ≤ 0.05, GemC18-SLNs (p.o.) vs. GemC18-free SLNs (p.o.) or Control; ^b^ *p* ≤ 0.05, GemC18-SLNs (p.o.) vs. GemC18 (p.o.) or GemHCl (p.o.); ^c^ *p* ≤ 0.05, Control vs. GemC18 (p.o.) or GemHCl (p.o.). In B, ^a-b^ indicate groups with the same letter are not different, while those with different letters are different on days 17 through 23, when the majority of the mice were still alive (*p* ≤ 0.05).

The oral antitumor activity of the GemC18-SLNs was further confirmed in mice with murine LLC lung tumors. Against LLC cells in culture (3 × 10^3^ cells, 48 h incubation), the IC_50_ values of gemcitabine HCl, GemC18, and GemC18-SLNs were 23.4 ± 6.7, 130.4 ± 31.1, and 159.4 ± 44.2 nM, respectively [40]. Similarly, oral GemC18-SLNs significantly inhibited LLC tumor growth in mice (Figure 3A). Both oral GemC18 (in 1% Tween 20) and oral gemcitabine HCl also inhibited LLC tumor growth, but they were significantly less effective than oral GemC18-SLNs at a molar equivalent dose (Figure 3A). Shown in Figure 3B are the body weight changes of the LLC tumor-bearing mice after they received various treatments. Oral GemC18-SLNs inhibited the growth of the mice (i.e. body weight increase as a function of time) as compared to untreated mice (i.e. 5% mannitol solution, p.o.), but the effect of the oral GemC18-SLNs on mouse body weight was not different from that of oral GemC18 or oral gemcitabine HCl (Figure 3B).

**Figure 4:**
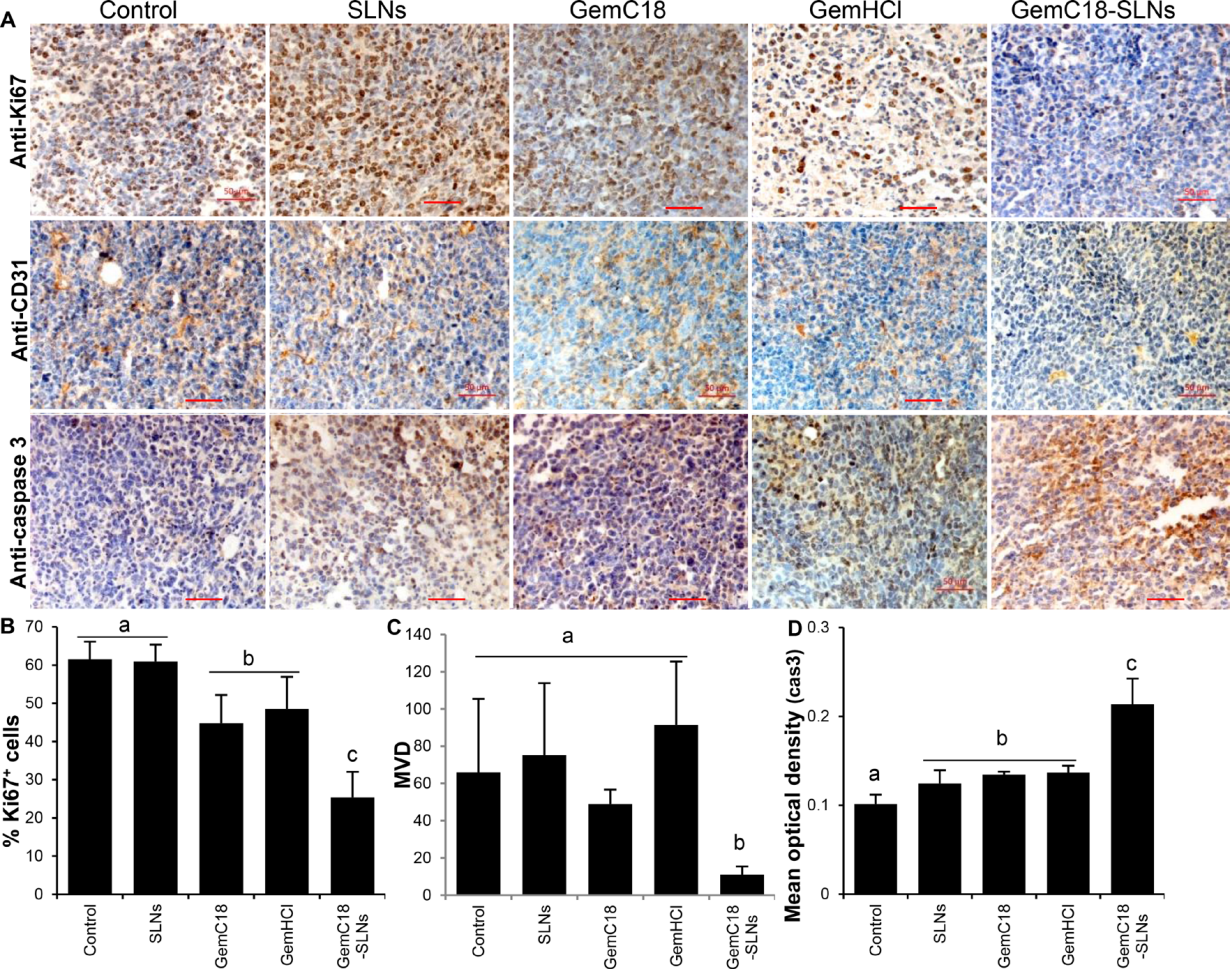
Immunohistostaining of LLC tumors from mice that were orally gavaged with GemC18-SLNs (**A**) Representative microscopic images of LLC tumor tissues after staining against Ki-67, CD31, or caspase 3. Mice were orally gavaged with GemC18-SLNs, gemcitabine HCl (GemHCl), GemC18 in Tween 20, GemC18-free SLNs, or a 5% mannitol solution. (**B**–**D**) Percent Ki67^+^ cells (B), microvessel density (MVD) (C), and percent caspase 3^+^ cells (D) in different tumor tissues. Data in B-D are mean ± S.D. from at least 3 different microscopic fields. In B-D, ^a-c^ indicate groups with the same letter are not different, while those with different ones are different (*p* ≤ 0.05).

Shown in Figure 4 are representative microscopic images of LLC tumor tissues from mice orally treated with GemC18-SLNs, GemC18 (in a Tween 20 solution), gemcitabine HCl, GemC18-free SLNs, or a 5% mannitol solution after the tumor tissues were stained with anti-Ki67 (a cell proliferation marker), anti-CD31 (an angiogenesis marker), or anti-caspase 3 (an apoptosis marker) antibodies. Anti-Ki67 staining revealed that tumor cell proliferation was significantly inhibited by the GemC18-SLNs (Figure 4A–4B). In addition, oral GemC18-SLNs also significantly inhibited angiogenesis in tumor tissues (Figure 4A, 4C) and induced more tumor cells to undergo apoptosis (Figure 4A, 4D).

Taken together, data in mice with either TC-1 or LLC lung tumors demonstrated that our GemC18-SLNs, when given orally, significantly inhibited tumor growth, and were more effectively than the molar equivalent dose of oral gemcitabine HCl or oral GemC18 alone (Figures 2–4). The mechanism underlying the strong oral antitumor activity of the GemC18-SLNs, relative to oral gemcitabine HCl, is likely related to the GemC18-SLNs’ ability to increase the oral bioavailability of gemcitabine (~70% for GemC18-SLNs vs. 10% in human subjects and 45% in FVB mice for gemcitabine [7, 32]). As mentioned above, it remains unknown how gemcitabine in the GemC18-SLNs was absorbed from the GI tract of mice. However, the observed increased oral bioavailability of gemcitabine from the GemC18-SLNs is likely related to the amide prodrug nature of the GemC18 and the SLNs in which the GemC18 was incorporated. Data from previous studies showed that other amide prodrugs of gemcitabine such as the LY2334737 and SQdFdC, when dosed orally in rodent models, showed stronger antitumor activity than the molar equivalent dose of gemcitabine [9, 23]. The systemic exposure (i.e. AUC) of oral LY2334737 in mice was also significantly higher than that of oral gemcitabine alone at a molar equivalent dose [9]. Therefore, the lipophilic amide prodrug nature of the GemC18 may have contributed to the increased oral bioavailability of the gemcitabine in the GemC18-SLNs [41, 42]. Of course, the PEGylated solid lipid nanoparticle nature of the GemC18-SLNs likely had also contributed to the strong oral antitumor activity of the GemC18-SLNs and the increased systemic exposure of gemcitabine after oral administration of GemC18-SLNs. It is thought that PEGylation of solid lipid nanoparticles can help the oral absorption of drugs incorporated in the nanoparticles by reducing mucin trapping, suppressing lipolysis, and/or improving mucosal permeability [43]. In addition, it was previously reported that the PEG chains allow nanoparticles of 200-500 nm to rapidly traverse mucosal barriers [44, 45]. Once permeating through the mucus layer, nanoparticles may permeate through the epithelial cell layer mainly via endocytosis by enterocytes or via uptake by the M cells [46]. Interestingly, Hu et al. (2015) reported that evidence does not support the absorption of intact solid lipid nanoparticles after oral administration in rodents [37]. The composition of our solid lipid nanoparticles is different from those used by Hu and coworkers in their study [37], and it is unknown whether, and to what extent, our solid lipid nanoparticles can be absorbed intact. Of course, as mentioned above, some GemC18 may be released from the GemC18-SLNs while in the GI tract, and is then absorbed as intact GemC18 or as gemcitabine after further hydrolysis. *In vitro* release of GemC18 from the GemC18-SLNs in SIF showed that less than 20% of the GemC18 was released within 6 h, but *in vivo*, lipases and other enzymes in the intestine may significantly affect the release of GemC18 from the GemC18-SLNs [26]. In future studies, we will monitor the release of GemC18 from the GemC18-SLNs in SIF that contains lipases and other enzymes to better estimate the rate at which GemC18 is released from the GemC18-SLNs in mouse intestinal tract, which will likely provide additional information about the mechanism of GemC18 absorption. Moreover, direct measurement of GemC18 concentration in mouse plasma samples after mice are orally gavaged with the GemC18-SLNs is expected to provide us additional information on the mechanism of GemC18 absorption. Of course, toxicity tests and more efficacy studies will have to be completed to determine the feasibility of translating the GemC18-SLNs into clinical trials, but knowledge learned with the GemC18-SLNs will likely help us design improved nanoparticle-based oral dosage forms of gemcitabine and/or other nucleoside analogs.

## MATERIALS AND METHODS

### Chemicals and cell lines

Mannitol, methanol (HPLC-grade), ethyl acetate (EtOAc), dichloromethane (DCM), potassium phosphate monobasic, sodium chloride, sodium hydroxide, Tween 20, GMS, and Tween 80 were from Sigma-Aldrich (St. Louis, MO). Gemcitabine HCl was from the U.S. Pharmacopeia (Rockville, MD) or Biotang, Inc. (Lexington, MA). Soy lecithin was from Alfa Aesar (Ward Hill, MA). DSPE-PEG_2k_ was from Avanti Polar Lipids, Inc. (Alabaster, AL). TC-1 mouse lung cancer cell line was from the American Type Culture Collection (Manassas, VA). TC-1 cells were grown in RPMI 1640 medium supplemented with 10% (v/v) fetal bovine serum (FBS), 100 U/mL of penicillin, and 100 μg/mL of streptomycin, all from Invitrogen (Carlsbad, CA). LLC mouse lung cancer cell line was from the Cell Bank of the Chinese Academy of Sciences (Shanghai, China). LLC cells were grown in DMEM medium supplemented with 10% (v/v) of FBS, 100 U/mL of penicillin, and 100 μg/mL of streptomycin, all from Biological Industries (Kibbutz Beit-Haemek, Israel).

### Preparation of 4-(*N*)-GemC18-SLNs

GemC18 was synthesized and GemC18-SLNs were prepared as previously described [26]. Briefly, 3.5 mg soy lecithin, 0.5 mg of GMS, DSPE-PEG_2k_ (11.6%, w/w, of total lipids and Tween 20), and 5 mg of GemC18 were placed into a 7 mL scintillation glass vial. One mL of de-ionized water was added into the mixture, which was then maintained on a 75°C hot plate while stirring, with occasional water-bath sonication (Bransonic Ultrasonic Cleaner, Danbury, CT), until the formation of a homogenous slurry. Tween 20 was added in a step-wise manner to a final concentration of 1% (v/v). The resultant emulsions were allowed to cool to room temperature while stirring to form nanoparticles. As a control, GemC18-free SLNs were prepared similarly but without GemC18. The size and zeta potential of the nanoparticles were measured using a Malvern Zetasizer Nano ZS (Westborough, MA). GemC18 was also dissolved in vegetable oil (ConAgra Foods, Omaha, NE) (GemC18-in-oil) or in Tween 20 (1%, v/v) in a 5% (w/v) mannitol solution. GemC18-SLNs and GemC18-free SLNs were used as freshly prepared or reconstituted from the lyophilized powders of the nanoparticles immediately before administration. The nanoparticles were lyophilized in the presence of 3% (w/v) of sucrose using a freeze-dryer from Labconco (Kansas City, MO). The particle size of the nanoparticles after reconstitution was not significantly different from that of the freshly prepared nanoparticles (*p* = 0.19, *t*-test, two-tailed, data not shown).

### Stability of the GemC18-SLNs and the *in vitro* release of GemC18 from the GemC18-SLNs

To evaluate the stability of the GemC18-SLNs in stimulated gastrointestinal fluids, non-fed SGF and SIF were prepared following the US Pharmacopeia-National Formulary. Briefly, SGF (pH 1.2) was prepared by dissolving 2 g of NaCl into 7 mL HCl, and the volume was adjusted to 1000 mL with deionized water. The SIF (pH 6.8) was prepared by adding 6.8 g of KH_2_PO_4_ and 896 mg NaOH into 1000 mL of deionized water. The GemC18-NPs were incubated in SGF or SIF at 37°C for 0, 1, 2, 4, and 6 h, and the particle size was measured using a Malvern Zetasizer Nano ZS.

To evaluate the *in vitro* release of GemC18 from the GemC18-SLNs in SGF and SIF, GemC18-SLNs in SGF or SIF were placed into a 1-mL cellulose ester dialysis tube (MWC, 50,000) from Spectrum Chemicals & Laboratory Products (New Brunswick, NJ). To make sure that the diffusion of the GemC18 across the dialysis tube membrane was not rate-limiting, GemC18-in-Tween 20 was also included. The dialysis tube was then placed into a plastic conical tube containing 13 mL of SGF or SIF with 0.05% Tween 80 and incubated in a 37°C shaker incubator. At predetermined time points (i.e. 0, 0.5, 1, 1.5, 2, 3, 4, or 6 h), 200 μL of the release medium was withdrawn and then immediately replaced with 200 μL of fresh release medium. The concentration of the GemC18 in the samples was determined by measuring the absorbance at 248 nm using a BioTek Synergy™ HT Multi-Mode Microplate Reader (Winooski, VT) [26].

### Plasma pharmacokinetics of gemcitabine

For all the animal studies, protocols were approved by the Institutional Animal Care and Use Committee (IACUC) at The University of Texas at Austin or the Biomedical Research Ethics Committee at the Inner Mongolia Medical University. To evaluate the pharmacokinetic parameters of gemcitabine when GemC18-SLNs were given orally or intravenously, healthy female BALB/c mice (Charles River, 6-8 weeks) were dosed with GemC18-SLNs (i.e. 1.0 mg of GemC18, equivalent to 0.497 mg of gemcitabine) by oral gavage (p.o.) or intravenous (i.v.) injection. At pre-determined time points (0.25, 0.5, 1, 2, 4, 8, 12, 24, or 48 h), mice were euthanized, and blood was collected into heparin-coated tubes and centrifuged (8000 x rcf) for 10 min to isolate plasma. Samples were stored at −80°C until analysis. A hydrolysis method was used to detect the total concentration of gemcitabine in plasma [38]. Briefly, to 75 μL of plasma sample, 25 μL of uracil 1-β-D-arabinofuranoside solution (AraU, 10 mg/mL) was added as an internal standard, followed by the addition of 100 μL of 2 N NaOH. This mixture was then vortexed and incubated at 40°C for 1 h. Following incubation, 800 μL of acetonitrile and 75 μL of 1.4 M H_3_PO_4_ was added, followed by centrifugation. The supernatant was then collected and dried under vacuum. Lastly, the residue was re-dissolved in 100 μL of PBS (pH 7.4, 2.5 mM) and centrifuged to collect the supernatant, which was then analyzed using an Agilent HPLC with an Agilent C18 column (5 μm, 4.6 mm × 250 mm; Santa Clara, CA) to measure gemcitabine concentration. The mobile phase was 5 mM sodium acetate (pH 6.0) and methanol (95/5, v/v), and the detection wavelength was 266 nm. Data were analyzed using the PK Solver^®^ and two-compartmental model [47].

### Evaluation of the antitumor activity of GemC18-SLNs in tumor-bearing mouse models

When TC-1 tumor cells were used, female C57BL/6 mice (18-20 g, 6-8 weeks, *n* = 5) were subcutaneously (s.c) injected with TC-1 cells (5 × 10^5^ cells/mouse) in the right flank on day 0. Mouse hair was carefully trimmed at the injection site one day prior to the injection. Treatment with GemC18-SLNs, GemC18-in-vegetable oil (GemC18-in-oil), GemC18-free SLNs, all in a mannitol solution (5%, w/v), was started on day 11, and mice were orally gavaged daily until the endpoint (i.e. death, tumor size reaching 15 mm, tumor ulceration, body weight loss of more than 20%, or other signs of severe distress and discomfort). As controls, mice were orally gavaged with a mannitol solution (5%) or i.v. injected with the GemC18-SLNs, twice a week until the endpoint. The dose of GemC18 was 250 μg per mouse per dose for the oral route, 500 μg per mouse per dose for i.v. route. Tumor size was measured 2-3 times a week, and tumor volume was calculated as: volume (mm^3^) = (length x width^2^)/2. Mouse survival time was also recorded. In another study, TC-1 tumor-bearing female C57BL/6 mice were orally gavaged with GemC18-SLNs, GemC18-in-oil, vegetable oil alone (oil), or gemcitabine HCl (GemHCl, as a control), once daily. The dose of GemC18 was 150 μg per mouse per dose, and dose of GemHCl was 85 μg per mouse per dose (i.e. molar equivalent to the dose of GemC18).

LLC tumors were established in female C57BL/6J mice (6-8 week) from the Beijing Vital River Laboratory Animal Technology Co., Ltd. (Beijing, China). Mice were s.c. injected with LLC cells (5 × 10^5^) in their back on day 0. On day 11, mice were randomized into groups (*n* = 8) and were treated by oral gavage with GemC18-SLNs, GemC18-free SLNs, GemC18 alone in a Tween 20 solution, GemHCl (molar equivalent of GemC18), all in a mannitol solution (5%, w/v). Control mice were orally gavaged with a mannitol solution (5%). Mice were treated once in every two days for 3 weeks. The dose of GemC18 was 250 μg/mouse, 141.5 μg/mouse for GemHCl (i.e. molar equivalent to 250 μg of GemC18). GemC18 was dissolved into 1% (v/v) Tween 20 in a 5% (w/v) mannitol solution to a proper concentration. Mouse health was monitored daily, and weight recorded. Tumor size was measured every other day. Twenty-four hours after the last dose, mice were sacrificed to collect tumor tissues, which were weighed, fixed, sectioned, and stained with antibodies against Ki67 (a cell proliferation marker, Abcam, Shanghai, China), CD31 (an angiogenesis marker, BioLegend, Beijing, China), or caspase 3 (a cell apoptosis marker, Bioss, Beijing, China). Microscopic images taken at 200 x magnification were analyzed using an Image-Pro Plus software (Media Cybernetics, Inc., Shanghai, China) to determine the percent of cells that are Ki67 positive, the microvessel density (MVD) in tumor tissues, and the optical density of the anti-caspase 3 staining.

### Statistical analysis

Statistical analyses were completed by performing analysis of variance, followed by Fisher's protected least significant difference procedure. Mouse survival curves were compared using the Mantel-Cox log-rank method using Prism^®^ from GraphPad Software, Inc. (La Jolla, CA). A *p* value of ≤ 0.05 (two-tail) was considered significant.

## CONCLUSIONS

In the present study, we found that the 4-(*N*)-stearoyl gemcitabine solid lipid nanoparticles (GemC18-SLNs) previously developed in our laboratories, when given orally, significantly inhibited tumor growth in mouse models, likely because the GemC18-SLNs led to increased systemic exposure of gemcitabine in the mouse models.
